# 10.B. Round table: Shelter in place: lessons from policy making during the COVID-19 pandemic in three countries

**DOI:** 10.1093/eurpub/ckac129.628

**Published:** 2022-10-25

**Authors:** Organised by: University of Augsburg (Germany), Chair persons: Verina Wild (Germany), Angus Dawson (Australia)

## Abstract

The key aims of the workshop are: (1) to engage in early discussion of the findings of this timely project and gain critical feedback from the EUPHA audience, (2) discuss and debate with the audience comparable and contrasting experiences from across Europe. This workshop will outline and discuss results from a project, funded by the Volkswagen Foundation, about the experience of decision-making and the implementation of non-pharmaceutical public health measures (such as social distancing, school closures, lockdowns etc) in the early stage of the response to COVID-19 (the pre-vaccine phase) in three countries (Germany, Israel and Australia). The project combines both empirical research in the form of qualitative interviews with key stakeholders as well as theoretical and conceptual ethical analysis. These methods are integrated to propose a comprehensive ethics assessment building upon the resources of public health ethics to improve the capacity for ethically sensitive decision-making in the future. Key objectives will be: (1) to record some of the relevant values that are explicitly appealed to by stakeholders, (2) to record any implicit values visible in the decision-making process, (3) discuss some of the value conflicts that arose during decision-making, (4) tentatively suggest lessons to be taken into account in future pandemic policy making. The format of this workshop will be a roundtable with a series of short (5 minute) presentations from five panellists, all part of the project, as set out below (25 mins). The chair will then invite questions on matters of clarification arising (max. 10 mins) before inviting members of the audience to provide short comments documenting and reflecting upon their similar or contrasting experiences in their countries (20 mins) before a brief conclusion (5 mins).

**Key messages:**

• Ethical values are implicitly or explicitly used to justify proposed and actual policy choices, even during a pandemic. Discussion of such values is useful for refinement of future pandemic policy.

• It was common to assume that everyone had the same access to information and ability to respond etc. However, a great, more explicit focus on disadvantaged or vulnerable groups would be beneficial.

